# VIOLENCE FROM PATIENTS AND VISITORS IN THE EMERGENCY DEPARTMENT – REPORTING OF AGGRESSION BY MEDICAL STAFF

**DOI:** 10.13075/ijomeh.1896.02640

**Published:** 2025

**Authors:** Anna Małgorzata Burak, Ewa Teresa Kamińska, Kamila Katarzyna Szulc, Patryk Krzewiński, Przemysław Żuratyński

**Affiliations:** 1 Nicolaus Copernicus University in Toruń, Collegium Medicum in Bydgoszcz, Faculty of Health Sciences, Department of Emergency Medical Services, Toruń, Poland; 2 University Hospital No. 2 in Bydgoszcz, Emergency Department, Bydgoszcz, Poland

**Keywords:** aggression, emergency department, violence at workplace, PVV, patient and visitor violence, reporting violence

## Abstract

**Objectives:**

Most hospital emergency department (ED) medical staff experience violence from patients and visitors every day, but the level of reporting and documenting acts of violence is very low. The aim of the study was to analyze the reporting of violence from patients and visitors by hospital ED medical staff.

**Material and Methods:**

The study was conducted retrospectively in the ED of a university hospital in Poland. The department admits 48 000 patients/year. The records kept by nurses and paramedics from 7 years (2017–2023) were analyzed for reports of acts of aggression from patients and visitors. The Python programming language was used for statistical analyses.

**Results:**

During the 7-year period, 445 cases of violence from patients and visitors were reported, with the number of reports decreasing significantly over the years. The average age of the aggressors was 47 years. Most of them were male (N = 318, 71.5%). Half of the aggressors were under the influence of alcohol (N = 218, 49%).

**Conclusions:**

The results suggest that not all incidents of violence are reported by staff and that their documentation may be influenced by various factors, both institutional and external. The descriptions of incidents of aggression are short and not very detailed, which makes it difficult to analyze their circumstances and nature. Effective prevention of aggression requires a thorough assessment of the scale and characteristics of this phenomenon. By reporting all incidents of aggression, staff can contribute to a reliable analysis of the problem and thus lead to increased safety in the workplace.

## Highlights

- Emergency departments are places with the highest rates of patient aggression toward healthcare workers.
- The level of reporting of violence by workers is low.
- Employees most often report only extreme cases of aggression.

## INTRODUCTION

Patient and visitor violence (PVV) is a global problem and is the subject of intense research [1–3]. Despite extensive analyses spanning over numerous years, and the implementation of various intervention strategies, the frequency and intensity of aggressive incidents have shown a persistent upward trend. Healthcare staff most often experience acts of emotional and verbal aggression [4–6]. This phenomenon has a significant adverse effect on their mental wellbeing [4] and simultaneously poses a threat to their safety, health and life. Sustained exposure to these conditions is a significant contributor to occupational stress, which may culminate in physical and psychological exhaustion [7]. Prolonged exposure to stress and negative emotions are conducive to experiencing professional burnout [8], the determinants of which include psychosocial and environmental factors as described in Puszczałowska-Lizis et al. [9]. The consequences of such processes do not only include a lowered sense of work satisfaction, but also ramifications such as depression, post-traumatic stress disorder (PTSD), and even resignation from work [3,5]. The COVID-19 pandemic further intensified the psychological strain on medical personnel, increasing the risk of fear, depression, PTSD and sleep disruption, particularly among the first line medical personnel responders such as emergency department (ED) staff [10,11].

Although emotional and verbal aggression dominate in terms of the number of incidents, physical aggression, while occurring less often, poses a direct threat to the health and life of staff. Research indicates that it affects a quarter of healthcare staff [5]. This form of aggression can result in physical injury and, in severe cases, fatality [1,5,12].

Reports from 2021 indicate that patient or visitor attacks have resulted in more than 370 fatal incidents of medical personnel, although the actual toll may likely be higher [12]. While extreme and fatal acts of aggression against medical personnel are often publicized, prompting public discourse on safety regulations and legal mechanisms, the true prevalence of this issue remains obscured by a pervasive underreporting. This is in direct contrast to long-standing calls from international bodies, such as the World Health Organization (WHO), to systematically monitor such incidents in order to inform and implement effective prevention protocols [13,14].

The results of numerous studies show that hospital EDs are among the settings with the highest PVV level [5,15–17], where violence against medical personnel is not a marginal occurrence, but rather a significant and recurring element of everyday professional practice [18]. Studies have identified a correlation between the environmental and structural conditions of these departments and an increased prevalence of aggression [19,20]. Researchers identify several key factors that contribute to this phenomenon, including the 24/7 accessibility of EDs, high patient volume [21], extended wait times, patient frustration, and inadequate safety systems [22]. Xie et at. [23] highlight that overcrowding contributes to aggressive patient behavior, especially when patients feel unfairly treated after being categorized with lower admission priority. Another predisposing factor for PVV is the involvement of medical personnel in tasks necessitating close personal contact [15]. Moreover, evidence suggests a correlation between the duration of direct close patient contact and the likelihood of experiencing aggression: the longer the interaction, the greater the probability of PVV [24]. All of these factors lead to the occurrence of aggressive behaviors targeted at medical personnel. Furthermore, patients that are under the influence of alcohol and with mental disorders are often responsible for aggressive behaviors [25].

However, despite the majority of ED personnel experiencing acts of aggression in their work, those who decide to record such acts are few [26]. Research points to different causes for PVV underreporting: the lack of clearly established procedures [15], the fear of professional repercussions, or the perception that such incidents are an inherent part of healthcare work [15,27,28]. This situation leads to a never-ending cycle, in which the lack of data regarding aggression makes it impossible to develop and implement an effective staff safety policy and appropriate training programs with special attention to ED work conditions.

### Aim of the study

The aim of the study was to determine the extent to which medical personnel report the aggressive behavior of patients and their visitors in the ED of the hospital. The description specificity of reported instances of aggression was analyzed, and potential challenges and obstacles related to this process were identified. The following research questions guided this study: to what extent is physical and verbal violence by patients documented by medical staff and how descriptive are nursing reports regarding incidents of aggression? What are the most frequently identified triggers for patient aggression according to medical personnel? What potential factors (e.g., perpetrator gender, presence of psychoactive substances, or behavioral disorders) are associated with different types of aggression? What is the frequency of intervention by hospital security or police in cases of aggression, and is physical restraint used? How did the COVID-19 pandemic affect the number and nature of reported aggression cases?

## MATERIAL AND METHODS

The study was a retrospective analysis conducted within the ED of a university hospital in the central part of Poland, in the city of Bydgoszcz. The department admits approx. 48 000 patients/year. A detailed review of nursing report books covering a 7-year period (2017–2023) was performed. During the study period 445 entries related to aggression were identified. Entries made by medical staff that referred to acts of aggression by patients and visitors were identified and extracted. They were classified as aggression-related if they contained clear references to aggressive behaviors exhibited by patients or visitors (e.g., physical violence, threats, insults, property damage). For the purpose of qualitative analysis, identified aggressive behaviors were assigned codes which were subsequently entered into an Excel spreadsheet. Coding was carried out independently by 2 researchers, ensuring the reliability and credibility of the analytical process. Any discrepancies identified were resolved by means of discussion. The study was approved by the Bioethics Committee at the Nicolaus Copernicus University in Toruń, Poland, KB 136/2024.

The Student's t-test was used for independent samples to describe the patient age variable. A normal distribution was assumed for all study subgroups, except for patients with mental disorders, due to the large sample size. In the subgroup of patients with mental disorders, the normality of the age distribution was assessed using the Shapiro-Wilk test (p = 0.399). Analyses also included verification of the assumption of equal variances (homogeneity), and if this assumption was not met, Welch's correction was automatically applied. If the expected subgroup size was not met, the Fisher-Freeman-Halton test was used. The χ^2^ test of independence was used to describe nominal variables. The statistical analyzes used the Python programming language (v. 3.11.6) with the following libraries: Pandas (v. 2.2.2), NumPy (v. 1.23.5), TableOne (v. 0.9.1), Matplotlib (v. 3.9.0), Seaborn (v. 0.13.2). All analyzes were performed assuming a significance level of 0.05.

## RESULTS

For this analysis, a total of 445 reports were collected in 2017–2023. These reports consisted of the following: 362 acts of verbal aggression (81.3%), 19 acts of physical aggression (4.3%), and 64 acts of both physical and verbal aggression (14.4%). For the reason that both types of aggression were denoted in a portion of the reports, their total number exceeds the number of reports including a certain type of aggression. Patients were often found to be the aggressors (86.5%). Men accounted for the majority of aggressive behaviors observed (Table 1). They were also significantly more likely to exhibit aggressive behavior than women (92.1% vs. 72.4%, p < 0.001) (Table 2). The average age of the aggressors was 47 years. Notably, the age of aggressive men was lower than that of aggressive women (M = 45.1 years vs. M = 52.3 years, p = 0.001). Descriptions of the causal factors for aggressive behavior were present in a subset of the reports. Among the causes, a lengthy waiting time was the most observed (N = 55, 12.4%). Aggressive behavior based on this premise was more often exhibited by women (23.6% vs. 7.9%, p < 0.001). The police intervened in 62 cases (14.6%). The use of direct coercion was significantly higher against men compared to women (17% vs. 4.7%, p < 0.001).

**Table 1.** Summary of patient and visitor violence incidents against emergency department staff at the University Hospital in Bydgoszcz, Poland, 2017–2023

Variable	Participants (N = 445)		M±SD
	n	%	
Demographic characteristics			
age [years]			47.1±18.9
gender			
female	127	28.5	
male	318	71.5	
Aggressive individual			
patient	385	86.5	
patient and family	13	2.9	
family	47	10.6	
Psychoactive substances			
alcohol	218	49.0	
other	39	8.8	
Mental disorder/behavioral disorder	23	5.2	
Incident details			
direct coercion	62	13.9	
voluntary departure	100	22.5	
Aggression			
type			
verbal	362	81.3	
verbal and physical	64	14.4	
physical	19	4.3	
physical aggression			
total	83	18.7	
most common conduct cited^a^			
tussling	26	5.8	
kicking	17	3.8	
damaging/destroying property	28	6.3	
attacking the staff	21	4.7	
not specified	17	3.8	
verbal aggression			
total	426	95.7	
most common conduct cited^a^			
vulgarities	146	32.8	
insults	93	20.9	
threats	43	9.7	
screaming	50	11.2	
issuing demands	18	4.0	
not specified	192	43.1	
belittling	60	13.5	

a The reports indicated >1 type of aggressive behavior.

**Table 2.** Selected report elements with a break down by gender of patient and visitor violence incidents against emergency department staff at the University Hospital in Bydgoszcz, Poland, 2017–2023

Variable	Participants (N = 445)									Test statistic value^a^	p
	total		M±SD	women (N = 127)		M±SD	men (N = 318)		M±SD		
	n	%		n	%		n	%			
Age [years]			47.1±18.9			52.3±21.3			45.1±17.5	–3.26	**0.001**
Aggressive individual										30.35	**<0.001**
patient	385	86.5		92	72.4		293	92.1			
family	13	2.9		7	5.5		6	1.9			
patient and family	47	10.6		28	22.0		19	6.0			
Psychoactive substance use											
alcohol										29.16	**<0.001**
no	227	51.0		91	71.7		136	42.8			
yes	218	49.0		36	28.3		182	57.2			
other										6.06	**0.014**
no	406	91.2		123	96.9		283	89.0			
yes	39	8.8		4	3.1		35	11.0			
Mental disorder/behavioral disorder										0.25	0.614
no	422	94.8		122	96.1		300	94.3			
yes	23	5.2		5	3.9		18	5.7			
Intervention											
security										0.12	0.734
no	427	96.0		123	96.9		304	95.6			
yes	18	4.0		4	3.1		14	4.4			
police										1.45	0.229
no	380	85.4		113	89.0		267	84.0			
yes	65	14.6		14	11.0		51	16.0			
Direct coercion										11.51	**0.001**
no	383	86.1		121	95.3		262	82.4			
yes	62	13.9		6	4.7		56	17.6			
Voluntary departure										2.31	0.129
no	345	77.5		105	82.7		240	75.5			
yes	100	22.5		22	17.3		78	24.5			
Aggression type										8.30	**0.016**
verbal	362	81.3		114	89.8		248	78.0			
physical	19	4.3		3	2.4		16	5.0			
both	64	14.4		10	7.9		54	17.0			

Bolded values are statistically significant.

a Value of t-statistic in case of Student's t-test, χ^2^-statistic for χ^2^ independence test.

This disparity may be attributed to a higher incidence of physical aggression among men, which was often accompanied by verbal aggression (17% vs. 7.9%). Of the total aggressive patient population, 22.5% were discharged from the hospital voluntarily (Table 1). The most frequently documented forms of verbal aggression were vulgarism (32.8%) and insulting medical personnel (20.9%). A significant gender-based difference in verbal aggression was observed. Men were more likely to use vulgarisms (37.4% vs. 21.3%, p < 0.001), while women more frequently issued demands (10.2% vs. 1.6%, p < 0.001). It is important to note that the specific characteristics of aggression were unspecified in nearly half of the reports, with the phrase “aggressive patient” being used without further detail. The most common physical aggressive behaviors described were damaging and destroying property (6.3%) and having physical altercations with medical personnel (5.8%) (Table 1).

Alcohol consumption was a notable factor in the analyzed cases of aggression. Nearly half of the aggressive individuals (N = 218, 49%) were under the influence of alcohol. This group consisted more of patients rather than visitors (96.3% vs. 3.7%, p < 0.001), and they were, on average, younger than their sober counterparts (43.9 vs. 50.4 years, p < 0.001). Alcohol-involved aggression was also significantly more prevalent in men (83.5% vs. 16.5%, p < 0.001). A lack of detailed behavioral descriptions for this subgroup was observed for a high percentage of reports simply noted an “aggressive patient under the influence of alcohol” (51.4% vs. 35.2%, p < 0.001). This lack of detail may explain why no complaints about medical care were formally recorded for this group. Despite this, a small portion of them did express complaints regarding waiting times (3.7% vs. 20.7%, p < 0.001). The behavioral profile of this group also showed some unique characteristics. They were less likely to scream (6.4% vs. 15.9%, p = 0.003) and did not issue demands (p < 0.001). Interventions were more frequent for this group, as police were called in a significantly higher percentage of cases (21.1% vs. 8.4%, p < 0.001). They also voluntarily departed the ED more often than sober patients (28.4% vs. 16.7%, p = 0.004).

Individuals under the influence of psychoactive substances other than alcohol were involved in 39 instances of aggression, representing 8.8% of the total cases. This group was characterized by a significantly younger mean age (29.6 years) compared to other aggressive individuals (48.9 years, p < 0.001). They were also predominantly male (89.7% vs. 69.7%, p = 0.014). In these cases, physical coercion was applied at a significantly higher rate (43.6% vs. 11.1%, p < 0.001). The use of physical aggression was also more frequent in this subgroup (38.5% vs. 16.7%, p = 0.002), specifically in the form of physical altercation (17.9% vs. 4.7%, p = 0.004). Additionally, reports of unspecified verbal aggression were more common for this group (66.7% vs. 40.9%, p = 0.003).

Aggression involving individuals with diagnosed mental or behavioral disorders accounted for 5.2% of the cases. This group demonstrated significantly higher rates of physical aggression (43.5% vs. 17.3%, p = 0.004) and was more likely to be subjected to physical coercion (30.4% vs. 13%, p = 0.029) compared to patients without such disorders. Getting into physical altercations was also more frequently described as part of their behavior (17.4% vs. 5.2%, p = 0.038).

**Figure 1.**
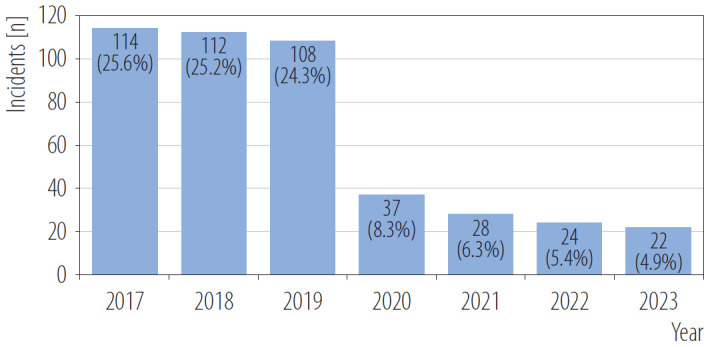
Reported patient and visitor violence (PVV) incidents against emergency department staff at the University Hospital in Bydgoszcz, Poland, in each year of the study, 2017–2023

A significant decline in the reported incidents of aggressive behavior was observed from 2020 onward, which may be related to the onset of the COVID-19 pandemic in Poland (Figure 1). From 2017 to 2019, a total of 334 aggressive incidents were documented, comprising 75.06% of all and corresponding to an annual average of approx. 2.3 incidents/1000 patients. In contrast, in 2020–2023, only 111 incidents were reported (24.94%), reducing the annual rate to approx. 0.6 incidents/1000 patients.

A comparison of aggression reports from 2017–2019 and 2020–2023 suggests that documentation in the latter period may have been limited to only the most severe incidents. Statistical analyses were conducted to investigate whether this reporting shift impacted the nature of the documented aggressive acts. The findings indicated a significant difference in the characteristics of aggressive incidents across the 2 time periods. Reports from the period 2020–2023 were almost exclusively related to aggressive patients (97.3% vs. 82.9%, p = 0.001), with a higher percentage of aggressors being men (79.3% vs. 68.9%, p = 0.047) (Table 3). Furthermore, the documented causes of aggression also changed. In the 2017–2019 period, a significant number of incidents were attributed to issues with healthcare or waiting times (15.9% vs. 1.8%, p < 0.001), but such causes were not reported in 2020–2023. This suggests that the process of recording the causes of aggression may have been discontinued in the latter period. The types of aggressive behaviors reported also shifted. In 2020–2023, there was a significant increase in the documentation of physical aggression: tussling (16.2% vs. 2.4%, p < 0.001), kicking (8.1% vs. 2.4%, p = 0.018), and property damage (10.8% vs. 4.8%, p = 0.042), as well as of verbal aggression: vulgarisms (43.2% vs. 29.3%, p = 0.01), insults (29.7% vs. 18%, p = 0.012), and belittling (21.6% vs. 10.8%, p = 0.006).

**Table 3.** Selected report elements of patient and visitor violence incidents against emergency department staff at the University Hospital in Bydgoszcz, Poland, divided into 2 periods 2017–2019 and 2020–2023

Variable	Participants (N = 445)									Test statistic value^a^	p
	total		M±SD	2017–2019 (N = 334)		M±SD	2020–2023 (N = 111)		M±SD		
	n	%		n	%		n	%			
Age [years]			47.1±18.9			47.1±19.1			47.0±18.2	–0.07	**0.946**
Gender										3.94	**0.047**
woman	127	28.5		104	31.1		23	20.7			
man	318	71.5		230	68.9		88	79.3			
Aggressive individual										16.925	**<0.001**
patient	385	86.5		277	82.9		108	97.3			
patient and family	13	2.9		13	3.9		0	0			
family	47	10.6		44	13.2		3	2.7			
Psychoactive substances											
alcohol										0.47	0.494
no	227	51.0		174	52.1		53	47.7			
yes	218	49.0		160	47.9		58	52.3			
other										2.14	0.144
no	406	91.2		309	92.5		97	87.4			
yes	39	8.8		25	7.5		14	12.6			
Mental disorders/behavioral disorders										3.47	0.063
no	422	94.8		321	96.1		101	91.0			
yes	23	5.2		13	3.9		10	9.0			
Healthcare										5.933	**0.015**
no	430	96.6		319	95.5		111	100			
yes	15	3.4		15	4.5		0	0			
Waiting time										13.95	**<0.001**
no	390	87.6		281	84.1		109	98.2			
yes	55	12.4		53	15.9		2	1.8			
Aggression											
type										21.29	**<0.001**
verbal	362	81.3		287	85.9		75	67.6			
physical	19	4.3		8	2.4		11	9.9			
both	64	14.4		39	11.7		25	22.5			
physical aggression											
most common conduct cited^a^											
tussling										26.47	**<0.001**
no	419	94.2		326	97.6		93	83.8			
yes	26	5.8		8	2.4		18	16.2			
kicking										5.93	**0.015**
no	428	96.2		326	97.6		102	91.9			
yes	17	3.8		8	2.4		9	8.1			
damaging/destroying property										4.15	**0.042**
no	417	93.7		318	95.2		99	89.2			
yes	28	6.3		16	4.8		12	10.8			
attack on medical personnel										0.81	0.369
no	424	95.3		316	94.6		108	97.3			
yes	21	4.7		18	5.4		3	2.7			
not specified										1.67	0.149
no	428	96.2		324	97.0		104	93.7			
yes	17	3.8		10	3.0		7	6.3			
verbal aggression											
most common conduct cited^a^											
vulgarisms										6.69	**0.010**
no	299	67.2		236	70.7		63	56.8			
yes	146	32.8		98	29.3		48	43.2			
insults										6.28	**0.012**
no	352	79.1		274	82.0		78	70.3			
yes	93	20.9		60	18.0		33	29.7			
threats										0.01	0.933
no	402	90.3		301	90.1		101	91.0			
yes	43	9.7		33	9.9		10	9.0			
screams										0.47	0.494
no	395	88.8		294	88.0		101	91.0			
yes	50	11.2		40	12.0		10	9.0			
issuing demands										1.715	0.190
no	427	96.0		318	95.2		109	98.2			
yes	18	4.0		16	4.8		2	1.8			
not specified										0.01	0.931
no	253	56.9		189	56.6		64	57.7			
yes	192	43.1		145	43.4		47	42.3			
belittling										7.49	**0.006**
no	385	86.5		298	89.2		87	78.4			
yes	60	13.5		36	10.8		24	21.6			

Bolded values are statistically significant.

a Value of t-statistic in case of Student's t-test, χ^2^ statistic for χ^2^ independence test, t-value for Fisher-Freeman-Halton test.^.^

Additionally, reports from 2020–2023 were significantly more likely to describe both verbal and physical aggression concurrently (p < 0.001). This, along with a significantly higher percentage of physical aggression overall (32.4% vs. 14.1%, p < 0.001), supports the hypothesis that only more extreme incidents were documented during this time. Despite this apparent increase in severity, the frequency of police or security intervention remained relatively constant across both periods (Table 3).

## DISCUSSION

This study is the first of its kind in Poland to feature an extensive observation period related to the recording of PVV. The results indicate a notably low level of reporting of instances of aggression, which likely does not reflect the actual magnitude of this phenomenon. Studies conducted in German hospitals resulted in findings consistent with the present study. Hahn et al. [15] indicated that a significant proportion of staff experienced verbal and physical violence; however, only a small percentage of incidents were formally reported. Similar observations were made by Benning et al. [18], who in their retrospective cohort study found that 859 incidents of PVV took place in the hospital ED in 2014–2023. According to the results of other studies, the phenomenon of medical personnel not reporting acts of violence is common [17,29]. In the context of international reports indicating that ≤90% of hospital ED staff experience different forms of violence [8,20], such a low level of reporting should raise concern and encourage an investigation of the causes of this phenomenon.

The results of this study corroborate earlier reports that aggression is most frequently used by patients and less often by their families or visitors [6,15,16]. Nursing and medical staff are met with the most extreme degree of aggression [5,6]. The dominant form of aggression encountered is verbal aggression [4,5,19], which is also reflected in the results of this study. Most often, patients were vulgar and insulted the staff. Liu et al. show that 42.5% of respondents experienced verbal violence in the form of insults or threats [5]. Other studies showed that verbal aggression most often encompasses screaming, insults, emotional aggression, vulgarisms [25,30] and undermining of the qualifications and threats of physical or sexual violence [4,31], and even death threats [4]. At the same time, the findings indicate that the terminology used by medical staff to describe aggressive behavior, particularly verbal aggression, may be inconsistent due to the lack of both standardized definitions and individual differences in the perception and interpretation of such behaviors. Reported actions such as “shouting,” “vulgarities,” and “insults” frequently co-occurred. However, during the coding process, they were treated as separate categories in order to maintain fidelity to the original documentation.

In the present study, physical aggression occurred less frequently than verbal aggression. In the studies by Liu et al. [5] as well as Doehring et al. [4], physical aggression accounted for 25% of cases. It was mainly expressed through damaging property and tussling with the medical personnel. Other researchers have also pointed to the following being used by patients against medical personnel: bumping, shoving, scratching, spitting, throwing objects, and hitting [16,17]; kicking, punching, biting, and spitting [4]; as well as, damaging glasses and uniforms of the medical personnel, threatening the use of dangerous tools against them [16], and ward infrastructure itself such as chairs or infusion stands [18].

Based on the analysis of the reports, it was determined that the most frequent causes for patient and visitor aggressive behavior were the following: the long waiting time for provision of medical care and the dissatisfaction with the quality of healthcare provided. Similar causes were indicated by other researchers [15,16].

This study showed that half of the aggressive individuals were under the influence of alcohol. A growing trend for aggressive individuals to be under the influence of other psychoactive substances is also evident. Researchers confirm that the individuals under the influence of psychoactive substances exhibit a higher probability of acting in an aggressive manner [32–34]. This is also true in the case of individuals with mental disorders [1,35]. The data indicate that an important risk factor for violence against the medical personnel, apart from the ED work circumstances, is the presence of individuals with disorders affecting cognitive processes. In this context, medical personnel often do not see the point to measures aimed at reducing PVV; they think of them as ineffective in light of the challenging working conditions and the specific characteristics of these patient groups [32].

Police and security interventions are not standard in acts of PVV. The most common approach is to ease the patient through conversation and only, in the case of aggression escalation, call the dedicated services [16]. Although medical personnel appreciate the role that law enforcement take in deescalating and increasing the overall sense of security [32], Frydrysiak et al. [28] indicate that calling police or security is treated as a last resort and occurs relatively rarely. Previous reports indicated, in turn, that 19% of respondents record only those instances of aggression where police are involved, which validates the claims of other researchers that medical personnel do not report all instances of aggression [27]. In Hahn et al.'s study only 1% of medical personnel used the official means of reporting acts of aggression, whereas one third recorded the acts of aggression in patient medical records [15].

The strategies for reducing the magnitude of PVV should encompass many aspects, including communication training and aggression handling. Despite the fact that the literature emphasizes these competencies, in practice these types of training are not widely conducted [15,32]. The personnel of the ED where the study was conducted participated in 2 training sessions in the area of patient communication in 2021–2024. The findings show that the effectiveness of such training is determined by a variety of factors, including the selection of the proper trainer and the participant's attitude to the educational process. A part of the staff, despite attending the training sessions, did not show readiness to implement the newly acquired tools for communication in daily clinical practice. Nonetheless, Xie et al. [23] emphasizes that especially in the case of emergency nurses, it is crucial not only to acquire knowledge and skills in their field of expertise, but also to develop the ability to foresee or anticipate and react appropriately to the incidents of violence, thereby realistically ensuring the safety of both medical personnel and patients. This is particularly relevant for younger nurses, who often signal lack of proper training in managing of PVV incidents [36].

The underreporting of aggressive acts by medical personnel is a result of a number of factors. One of the key factors is the belief that violence is an inseparable element of ED work, which leads to its normalization [19,37]. This belief is further reinforced by the systematic occurrence of PVV incidents, which are often not reported or documented in any formal fashion [32]. Consequently, the behaviors that should be deemed as unacceptable and should not be tolerated are accepted as part of the reality of the profession [17]. The misestimation of the magnitude of PVV fosters the ‘normalization” of violence as an inherent aspect of work, which subsequently reduces the willingness to report future incidents, thus creating a vicious cycle of concealment of the problem.

A 2021 study shows that medical personnel often perceive reporting aggressive behavior as pointless, which additionally adds to the underreporting of the issue [27]. The consequences of this misestimation result in a fallacious view regarding the phenomenon's magnitude, as well as hinder the ability to perform international comparisons and increase the risks of arriving at false conclusions as to the effectiveness of preventive measures. Consequently, departments such as EDs might not receive adequate staffing, infrastructure and training support.

Another significant factor that impacts the aggressive behavior reporting level is the engagement of the organization. The results of this study conducted in the facility showed a higher level of reporting acts of aggression in the first few years of the study. This phenomenon coincided with the hospital entering into an accreditation program for hospitals. For this reason, among others, a greater emphasis was placed on reporting PVV incidents, a shift reflected in the results of this study. Similar observations were reported by Richardson et al. [17], who confirms the major divergence in reporting aggression incidents in EDs in 2 different time intervals. During 1 of these periods, a campaign related to reporting aggressive acts was conducted, which led to a substantial increase in the level of their reporting in that period. These results indicate the significance of organizational actions in the shaping of medical personnel attitudes with regards to reporting aggression, and they highlight the need to implement system solutions that would support this process.

According to some reports from the COVID-19 pandemic period, as a result of the increased number of hospitalized patients, the organizational chaos, and disinformation, an increased number of aggressive acts towards personnel was recorded [38,39]. Brigo et al. [38] in a study conducted in an ED in 2017–2021 showed an almost 86-fold increase in attacks on medical personnel by patients. This study showed a significant decrease in reported PVV instances from 2020, coinciding with the onset of the COVID-19 pandemic in Poland. Reporting of PVV incidents became largely limited to cases of extreme aggression, as a result of which the number of reported acts of physical aggression significantly increased. At the same time, almost half of the reported incidents contained only abbreviated information on the acts of physical aggression, often limited to the fact that such acts simply occurred, which might indicate the marginalization of the PVV problem in the light of the pandemic and the increased workload of the medical personnel. Congruent observations were made by Benning et al. [18], who indicated that the reduction of PVV in 2020–2021 might have resulted not from an actual decline in aggression, but rather from limited PVV reporting, infrequent personnel training sessions and a reduced number of patients during the pandemic. Additionally, the psychological consequences of working in the pandemic conditions might have fostered a lowered resilience of medical personnel in the face of difficult or confrontational patient encounters. Although this interpretation appears plausible, it is yet to be confirmed by research. Nevertheless, the analysis of Benning et al. [18] as well as those of the present study show that the magnitude of PVV reporting after the pandemic has not returned to its pre-pandemic levels. In the context of the increased mental strain on the medical personnel during this time [10] the lack of reliable data regarding the extent of aggression makes it difficult to design appropriate interventive measures which would support staff mental wellbeing. Therefore, any hypothesis regarding the extent of influence of the pandemic on the reduced reporting must be formulated with caution.

The development and implementation of appropriate reporting mechanisms would make it possible to effectively control the risk regardless of how critical a situation would get. However, the most crucial aspect is the attitude of healthcare staff and reporting all cases of aggression. Furthermore, institutional and legal regulations should clearly protect healthcare workers by guaranteeing them safe working conditions and building a culture of safety within healthcare facilities that ensures both staff and patients will feel protected and respected.

### Limitations of the study

The study is subject to some limitations. First, the data come from a single university hospital in Poland, which admits approx. 48 000 patients to its ED annually. Second, the analysis of medical documentation was difficult due to the often laconic and imprecise form of information contained therein. A potential issue arises from the nature of study, and more specifically from the retrospective data acquired for the purposes of the study as well as the lack of standardization contained in the reports therein. Finally, another limitation is the fact that not all cases of aggression were reported by medical personnel, which may lead to an inaccurate estimate of the phenomenon's true prevalence.

## CONCLUSIONS

Emergency department staff experience violence from patients more often than from visitors. Most often, it is verbal aggression in the form of vulgarity and insults. Physical aggression is less common and usually results in property damage and direct attacks on medical staff. Most aggressive behavior is initiated by men. Half of those who exhibit aggressive behavior are under the influence of alcohol, but the number of incidents involving patients under the influence of other psychoactive substances is increasing. The level of reporting of aggressive incidents by staff is low. Their description is limited to a brief and general mention of the occurrence of aggression, which makes it difficult to conduct a full analysis process that would take into account the circumstances and nature of the aggression. The implementation of a standardized incident reporting form could facilitate an unambiguous classification of aggressive behaviors. This, in turn, would significantly enhance the consistency and accuracy of reporting, and improve further quantitative and qualitative analyses.
